# Esophageal Carcinoma with Triplicate Differentiation into Squamous Cell Carcinoma, Small Cell Carcinoma and Adenocarcinoma: a Case Report

**DOI:** 10.4021/gr2009.04.1282

**Published:** 2009-03-20

**Authors:** Tadashi Terada

**Affiliations:** Department of Pathology, Shizuoka City Shimizu Hospital, Miyakami 1231 Shimizu-Ku, Shizuoka 424-8636, Japan. Email: piyo0111jp@yahoo.co.jp

**Keywords:** Esophagus, Squamous cell carcinoma, Small cell carcinoma, Adenocarcinoma, Immunohistochemistry

## Abstract

Esophageal carcinoma with multiple differentiation is very rare. The author herein reports a case of esophageal carcinoma with triplicate differentiation (squamous cell carcinoma, small cell carcinoma, and adenocarcinoma). A 78-year-old man was admitted to our hospital because of dysphagia. An endoscopic examination revealed a polypoid tumor (3 x 4 x 3 cm) in the distal esophagus, and biopsy was obtained. The biopsy showed a tumor composed of moderately differentiated squamous cell carcinoma, small cell carcinoma, and adenocarcinoma. The proportions of them were 40% in squamous cell carcinoma component, 50% in small cell carcinoma component, and 10% in adenocarcinoma. There were gradual merges among them. Immunohistochemically, squamous cell carcinoma component was positive for cytokeratins and p53 protein. The Ki-67 labeling was 43%. The small cell carcinoma component was positive for cytokeratin, p53 protein, CD56, and KIT. The Ki-67 labeling was 95%. The adenocarcinoma component was positive for mucins, cytokeratin, p53 protein and CEA. The KI-67 labeling was 52%. The author speculates that this carcinoma arise from totipotent stem cell of the esophagus. The patient was treated by chemoradiation therapy, but died of systemic metastasis 13 months after the initial manifestation.

## Introduction

Esophageal carcinomas with multiple differentiation are very rare in the English literature [1-12]. Most of them are associated with small cell carcinoma of the esophagus. Basaloid sauamous cell carcinoma of the esophagus is also known to display multiple differentiation [13]. The author herein reports a very rare case of esophageal carcinoma with triplicate differentiation into squamous cell carcinoma, small cell carcinoma, and adenocarcinoma.

## Case report

**Figure 1:**
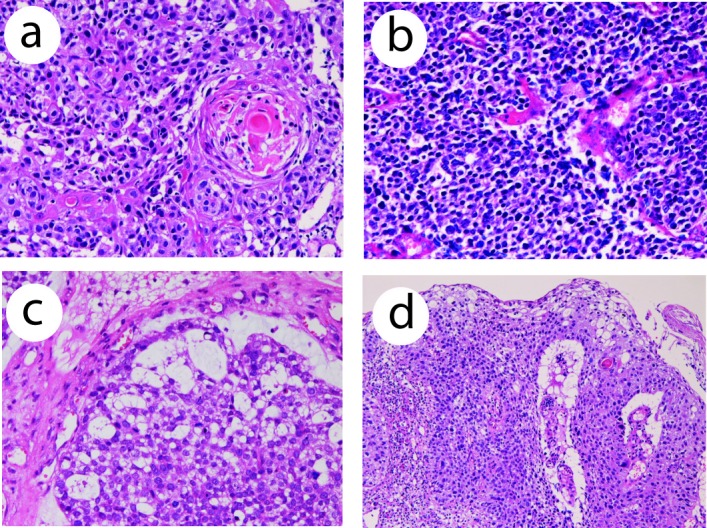
(a) Histology of squamous cell carcinoma element of the esophageal tumor. Keratinization is seen. HE, x 200. (b) Small cell carcinoma element of the esophageal carcinoma. The tumor cells shows characteristic morphologies of small cell carcinoma. HE, x 200. (c) Adenocarcinoma element of the esophageal carcinoma. Tubular formations are seen. There is a gradual transition between adenocarcinoma element and small cell carcinoma element. HE, x 200. (d) Transitional zone between squamous cell carcinoma element and small cell carcinoma element of the esophageal carcinoma. HE, x 100.

A 78-year-old man was admitted to our hospital because of dysphagia. An endoscopic examination revealed a polypoid tumor (3 x 4 x 3 cm) in the distal esophagus, and biopsy was taken. The biopsy showed a tumor composed of moderately differentiated squamous cell carcinoma (Fig. 1a), small cell carcinoma (Fig. 1b), and adenocarcinoma (Fig. 1c). The proportions of them were 40% in squamous cell carcinoma, 50% in small cell carcinoma, and 10% in adenocarcinoma. The squamous cell carcinoma element showed keratinization and intercellular bridge (Fig. 1a). The small cell carcinoma element was composed of malignant small cells with hyperchromatic nuclei, scant cytoplasm, finely granular chromatin, molded nuclei, and absent or inconspicuous nucleoli (Fig. 1b). The adenocarcinoma element was composed of malignant cells with tubular formations (Fig. 1c) with neutral and acidic mucins. There were gradual transitions among them (Fig. 1c, d).

**Figure 2:**
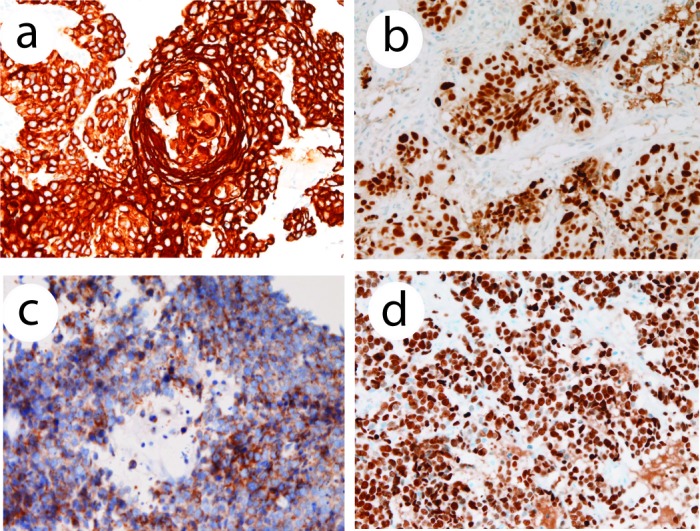
(a) High molecular weight cytokeratin is positive in the squamous cell carcinoma element. Immunostaining (AE1/3), x 200. (b) p53 expression in squamous cell carcinoma element of the esophageal tumor. Immunostaining, x 200. (c) Synaptophysin is positive in the small cell carcinoma element of the esophageal tumor cells. Immunostaining, x 200. (d) Ki-67 labeling in the small cell carcinoma element of the esophageal tumor. The labeling is 95%. Immunostaining, x 200.

An immunohistochemical study was performed by Dako Envision method (Dako Corp, Glostrup, Denmark), as previously described [14-17]. Immunohistochemically, the squamous cell carcinoma component was positive for pancytokeratins (AE1/AE3, polyclonal wide, Dako), high-molecular weight cytokeratin (34βE12, Dako) (Fig. 2a), cytokeratin (CK) 7, CK8, CK18, CK19, CK20, and p53 protein (Fig. 2b). The Ki-67 labeling (MIB1, Dako) was 43%. It was negative for CEA (Dako), CD56 (Dako), chromogranin (Dako), neuron-specific enolase (Dako), synaptophysin (polyclonal, Dako), vimentin, and KIT (polyclonal, Dako). The small cell carcinoma component was positive for pancytokeratins, CK7, CK8, CK18, p53 protein, synaptophysin (Fig. 2c), CD56, and KIT. The Ki-67 labeling was 95% (Fig. 2d). It was negative for high-molecular weight cytokeratin, CK19, CK20, neuron-specific enolase, CEA, vimentin, and chromogranin. The adenocarcinoma component was positive for pancytokeratins, CK7, CK8, CK18, CK19, p53 protein, and CEA. The Ki-67 labeling was 52%. It was negative for high molecular weight cytokeratin, CK 20, neuron-specific enolase, CD56, chromogranin, synaptophysin, vimentin, and KIT.

The patient was pathologically diagnosed as an esophageal carcinoma with triple differentiation (squamous cell carcinoma, small cell carcinoma, and adenocarcinoma). The patient was treated by radiation (50 Gray) and cisplatin-based chemotherapy, but died of systemic metastasis 13 months after the initial presentation

## Discussion

Histologically, the present esophageal tumor was composed of three elements. The pathology and immunohistochemistry were characteristic of each tumor. The squamous cell carcinoma component was characterized by keratinization, intercellular bridge and high-molecular weight cytokeratin, the small cell carcinoma component by characterized cellular morphologies and neuroendocrine antigens, and adenocarcinoma by tubular formations, mucin and CEA. Therefore, the diagnosis of the present tumor is correct.

Histologically, there were gradual transitions among the three elements. The proportions of these three elements were 40% in squamous cell carcinoma element, 50% in small cell carcinoma element, and 10% in adenocarcinoma element. The higher proportion of small cell carcinoma suggests that the sqaumous cell carcinoma element and adenocarcinoma element were differentiated from the small cell carcinoma element. Otherwise, this esophageal tumor arises from totipotent stem cell of the esophagus, as suggested by Ho et al [2].

Most of esophageal tumors with multiple differentiation is small cell carcinoma [1-12], although basaloid cell squamous cell carcinoma also shows multiple differentiation [13]. The cellular origin of small cell carcinoma is unknown. In the review of the English literature on multiple differentiation of esophageal cancers, Maru et al [1] reported that a combination of small cell carcinoma and adenocarcinoma was seen in 15/40 cases, and a combination of small cell carcinonoma and squamous cell carcinoma in 1/40 cases. Ho et al [2] reported that a combination of small cell carcinoma and squamous cell carcinoma or adenocarcinoma was seen in 3/4 cases. Sasajima et al [3] demonstrated one case of esophageal carcinoma showing multiple differentiation into oat cell carcinoma, adenoid cystic carcinoma, adenocarcinoma, and squamous cell carcinoma. Reyes et al [4] identified that small cell carcinoma coexisted with squamous cell carcinoma in 4/16 cases. Reid et al [5] showed a case of combination of small cell carcinoma and squamous cell carcinoma. Yun et al [6] identified squamous differentiation in small cell carcinoma in 2/21 cases. Medgyesy et al [7] found a combination of small cell carcinoma and adenocarcinoma in 1/8 case, and a combination of small cell carcinoma and squamous cell carcinoma in 1/8 case. Wu et al [10] reported that small cell carcinoma with squamous cell carcinoma was found in 3/9 cases. Yamamoto et al [11] demonstrated a combination of small cell carcinoma and squamous cell carcinoma was seen in 3/6 cases. Takubo et al [12] found a combination of small cell carcinoma and squamous cell carcinoma in 11/21 cases, and a combination of small cell carcinoma and mucoepidermid carcinoma in 1/21 cases. Finally, Cho et al [14] identified a combination of basaloid squamous cell carcinoma and squamous cell carcinoma in 8/18, a combination of basaloid squamous cell carcinoma and adenocarcinoma in 3/18 cases, a combination of basaloid squamous cell carcinoma and small cell carcinoma in 2/18 cases. The review of esophageal carcinoma with multiple differentiation is mostly limited to two differentiation. Esophageal carcinoma with more than three differentiation as seen in the present study, is extremely rare.

In summary, the author presented an extremely rare case of esophageal carcinoma with triplicate differentiation (squamous cell carcinoma, small cell carcinoma, and adenocarcinoma. The author speculates that the tumor is basically small cell carcinoma with squamous and adenocarcinomatous differentiation, or that the present esophageal carcinoma arises from totipotent stem cell of the esophagus.
